# Brown adipose tissues mediate the metabolism of branched chain amino acids during the transitioning from hyperthyroidism to euthyroidism (TRIBUTE)

**DOI:** 10.1038/s41598-022-07701-7

**Published:** 2022-03-07

**Authors:** Lijuan Sun, Hui Jen Goh, Sanjay Verma, Priya Govindharajulu, Suresh Anand Sadananthan, Navin Michael, Christiani Jeyakumar Henry, Julian Park-Nam Goh, S. Sendhil Velan, Melvin Khee-Shing Leow

**Affiliations:** 1 Singapore Institute for Clinical Sciences, Agency for Science, Technology and Research (A*STAR), 30 Medical Drive, Singapore, 117609 Singapore; 2 Laboratory of Molecular Imaging, Institute of Bioengineering and Bioimaging, Agency for Science Technology and Research (A*STAR), Singapore, Singapore; 3 Singapore Institute of Food and Biotechnology Innovation, Agency for Science Technology and Research (A*STAR), Singapore, Singapore; 4 Department of Biochemistry, Yong Loo Lin School of Medicine, National University of Singapore (NUS), Singapore, Singapore; 5 Department of Diagnostic Radiology, Tan Tock Seng Hospital (TTSH), Singapore, Singapore; 6 Departments of Physiology and Medicine, National University of Singapore (NUS), Singapore, Singapore; 7 Lee Kong Chian School of Medicine, Nanyang Technological University (NTU), Singapore, Singapore; 8 Department of Endocrinology, Tan Tock Seng Hospital (TTSH), Singapore, Singapore; 9 Yong Loo Lin School of Medicine, National University of Singapore, Singapore, Singapore; 10 Cardiovascular and Metabolic Disorders Program, Duke-NUS Medical School, Singapore, Singapore

**Keywords:** Metabolism, Thyroid gland

## Abstract

Both hyperthyroidism and elevated plasma branched chain amino acids (BCAA) are associated with insulin resistance. BCAA utilization and clearance relative to thyroid status changes remains unclear. We investigate amino acids changes, specifically BCAA, during the transition from hyperthyroidism to euthyroidism, and the impact of active brown adipose tissue (BAT) on the metabolic effects of BCAA. Newly diagnosed Graves’ disease participants were recruited. Hyperthyroidism was treated via a titration dosing regimen of thionamide anti-thyroid drug to establish euthyroidism over 12–24 weeks. All underwent energy expenditure (EE) measurement within a chamber calorimeter, ^18^F-fluorodeoxyglucose (^18^F-FDG) positron-emission tomography/magnetic resonance (PET/MR) imaging and plasma amino acids measurement during hyperthyroidism and euthyroidism. PET BAT maximum standardized uptake value (SUVmax), SUVmean and MR supraclavicular fat fraction (FF) quantified BAT activity. Twenty-two patients completed the study. Plasma BCAA level was significantly reduced in BAT-positive but not in BAT-negative patients during the transition from hyperthyroidism to euthyroidism. Plasma valine but not leucine and isoleucine correlated positively with insulin and HOMA-IR in hyperthyroidism. Plasma valine, leucine and isoleucine correlated with insulin and HOMA-IR in euthyroidism. Plasma valine correlated with insulin and HOMA-IR in BAT-negative but not in BAT-positive participants in both hyperthyroid and euthyroid state. However, the change (i.e. decrease) in plasma valine concentration from hyperthyroid to euthyroid state was affected by BAT-status. BAT utilizes and promotes BCAA plasma clearance from hyperthyroid to euthyroid state. Active BAT can potentially reduce circulating BCAA and may help to ameliorate insulin resistance and improve metabolic health.

Clinical trial registration: The trial was registered at clinicaltrials.gov as NCT03064542.

## Introduction

Brown adipose tissue (BAT) is a thermogenic organ. Besides its function in the regulation of energy expenditure (EE), activated BAT serves as a sink for the metabolic substrates, glucose and fatty acids. Given its lipid and glucose-lowering effects, there is a strong interest in BAT as a therapeutical target for improving metabolic syndrome^1,2^.

Thyroid hormone is an important determinant of EE and basal metabolic rate^3^. Thyroid hormone 3,5,3ʹ-triiodothyronine (T3) is also one of the key essential hormones that contribute to BAT activation and white adipose tissue (WAT) browning^4–6^. Chronic hyperthyroid patients are characterized by increased EE and activation of the sympathetic nervous system (SNS)^7,8^. BAT is activated by both thyroid hormones and sympathetic activity, which results in elevated BAT utilization of lipid/glucose substrates in hypertyroid patients^8^. As BAT metabolic activity is very high, even a small quantity of functional BAT can have a large effect on metabolism^9^.

Recently, Yoneshiro et al.^10^ reported that activated BAT serves as a metabolic-filter that controls branched chain amino acids (BCAA; valine, leucine and isoleucine) clearance. BCAA are essential amino acids which are thought to be beneficial to protein synthesis and energy expenditure^11^. However, paradoxically, elevated circulating BCAA levels have also been linked to insulin resistance and diabetes^12^. Hyperthyroid patients have altered muscle protein metabolism with increased protein breakdown. This contributes to muscle weakness/muscle loss as well as elevated circulating BCAA levels (4,13,14). The elevation in BCAA may be a compensatory mechanism to preserve muscle protein, since BCAA are known to stimulate protein synthesis and suppress protein breakdown^13^. The elevated basal insulin levels in hyperthyroid subjects may also be a compensatory mechanism to suppress muscle protein breakdown^14^. Therefore, hyperthyroidism can have a significant effect on glucose metabolism and the development of insulin resistance^15^.

Although active BAT has been thought to protect against the the development of insulin resistance partly by reducing circulating BCAA level, the complex interrelationships between BAT, BCAA and thyroid hormones merit further exploration. In this study, we aimed to evaluate the effects of activated BAT on BCAA catabolism during the transition from hyperthyroidism to euthyroidism consequent to medical therapy of Graves’ disease in South East Asian patients.

## Materials and methods

### Study participants

Participants between 21 and 65 years of age with newly diagnosed Graves’ disease attending the endocrine outpatient clinics of a local general hospital were recruited between October 2017 and June 2019. Graves’s disease was diagnosed based on laboratory and clinical evidence of primary hyperthyroidism and positive serum TSH receptor autoantibodies. They were enrolled if anti-thyroid drugs (ATD) (carbimazole/thiamazole) were not initiated for more than a month and if their latest thyroid function test (TFT) still reflected frank biochemical hyperthyroidism. Patients who were pregnant or contemplating pregnancy, allergic to carbimazole (CMZ), thiamazole (TMZ), taking medication which may affect body composition (eg. steroids) or BAT (eg. beta-blockers) or have a history of claustrophobia which hindered MRI scanning were excluded.

The study was conducted according to the ethical guidelines of the Declaration of Helsinki, and all procedures were approved by the Domain-Specific Review Board of National Healthcare Group, Singapore (Ethics code C/2015/00718) and registered with ClinicalTrials.gov (NCT03064542). Written informed consent was obtained from all subjects before participation.

### Study protocol

The patients participated in a screening visit to evaluate eligibility. Once the participants had consented to participate, they received standard ATD therapy using a decremental titration dosing regimen with CMZ or TMZ. The patients were subsequently scheduled for visit 2 for baseline research measurements while they were still hyperthyroid.

On study visit 2, patients went to the Clinical Nutrition Research Centre (CNRC) in the morning at 0830 h after an overnight fast of 8–10 h. All the patients underwent anthropometry (i.e. measures of body weight, height, waist and hip circumference) and body composition evaluation using dual energy X-ray absorptiometry (DXA) which measure fat, lean mass and bone mass. After finishing the measurements, they underwent metabolic rate measurement in a whole body calorimeter for 45 min. Then, they proceeded to the Clinical Imaging Research Centre (CIRC). Upon arrival in CIRC, an indwelling intravenous cannula was inserted into a forearm vein by a phlebotomy-trained state registered nurse and a baseline blood sample was obtained. The patients were then given an intravenous injection of a radiolabeled glucose (^18^F-FDG) through the intravenous cannula and followed by fusion PET-fat fraction MRI scanning for BAT and MRI-MRS of abdominal white fat for the next 1 h.

After completion of visit 2 procedures, the patients were required to continue with follow-up at the endocrinology clinic for control of their hyperthyroidism. Clinic follow up reviews occurred at 6–8 weekly intervals as per standard medical practice and ATD doses titrated against thyroid function tests and symptoms till euthyroidism was achieved. This took up to about 6 months. Subsequently, the patients continued with study visit 3 in exactly the same fashion as study visit 2.

### Clinical measurements

Fat mass, bone mass and lean mass were measured by dual energy X-ray absorptiometry (DXA) (QDR 4500A, fan-beam densitometer, software version 8.21; Hologic, Waltham, USA). Body composition measurements were performed in all study participants at baseline (during hyperthyroidism) and after achieving euthyroidism.

Resting energy expenditure (REE) was assessed through gaseous exchanges using a dual-chamber whole body calorimeter (WBC) facility located at the Clinical Nutrition Research Centre (CNRC). The WBC has been described in detail in our previous published paper^16^. REE was measured before and after intervention. Fasting glucose, insulin, total cholesterol, high-density lipoprotein (HDL) cholesterol, low-density lipoprotein (LDL) cholesterol, triglyceride, free T4 (FT4) and free T3 (FT3) were determined by National University Hospital referral laboratory.

All PET and MR scans were performed by using a hybrid PET-MR system (Biograph mMR, Siemens Healthcare, Erlangen, Germany) for 60 min. Intravenous injection of ^18^F-FDG (3 mCi) was administered, 60 min before imaging. A graph cut algorithm was utilized for fat–water separation using the International Society for Magnetic Resonance in Medicine (ISMRM) fat–water Toolbox^17,18^. Supraclavicular fat fraction (sBAT) depots were manually segmented based on anatomical information in multiple slice images of registered MR and PET images using Insight Segmentation and Registration Toolkit SNAP (ITK-SNAP) under the close guidance of an experienced clinical radiologist^19^. A lower threshold of 40% of sFF values was used to exclude the muscle and bone marrow prior to computation of mean MR FF, PET SUV mean and PET SUV max in the segmented sBAT region^20,21^. The cut-off value of sBAT PET SUV max for categorizing subjects into BAT-positive and BAT-negative groups was 1.5^22^.

MR images of abdominal fat were acquired using 2-point Dixon on the hybrid MR-PET system (Biograph mMR, Siemens). A fully automated graph theoretic segmentation algorithm was used to separate and quantify the visceral adipose tissue (VAT) depots^23,24^.

### Amino acids measurements

50 µL of serum samples were extracted using methanol and dried under nitrogen gas. The dried extracts were derivatised with 3 M hydrochloric acid in butanol (Sigma Aldrich, USA) and diluted in water for analysis via liquid chromatography-mass spectrometry (LC–MS). Deuterated stable isotopes of the respective amino acids were used as internal standards. Targeted analysis of amino acid was performed by LC–MS. LC–MS analysis were conducted on an Agilent 1290 Infinity LC system (Agilent Technologies, CA, USA) coupled with quadrupole-ion trap mass spectrometer (QTRAP 5500, AB Sciex, DC, USA). The samples were separated using a C18 column (Phenomenex, 100 × 2.1 mm, 1.6 μm, Luna® Omega). Mobile phase A (Water) and Mobile phase B (Acetonitrile) both containing 0.1% Formic acid were used for the chromatography separation. The LC run was performed at a flow rate of 0.4 mL min^−1^ with initial gradient of 2% B for 0.8 min, then increased to 15% B in 0.1 min, 20% B in 5.7 min, 50% B in 0.5 min, 70% B in 0.5 min, followed by re-equilibration of the column to the initial run condition (2% B) for 0.9 min. All compounds were ionized in positive mode using electrospray ionization. The chromatograms were integrated using MultiQuant™ 3.0.3 software (AB Sciex, DC, USA). The serum amino acids (AA) was measured in Duke-NUS Metabolomics facility, Singapore.

### Statistical analysis

Differences between BAT-positive and BAT-negative groups were assessed using Student’s t-test. Paired-t tests were used to compare the differences in the parameters between baseline and after intervention. The linear mixed effects model was used to evaluate the main effects of patient status effects (hyperthyroidism and euthyroidism), BAT status effects (BAT-positive and BAT-negative) as well as their interactions after adjusting for BMI. The primary endpoint was a reduction of BCAA during the transition from hyperthyroidism to euthyroidism. Spearman correlations were used to assess relationships between variables. Data are presented as means ± SEM, unless otherwise stated. Statistical analysis was performed by using SPSS software version 23 (IBM SPSS Inc.).

## Results

### Clinical measurements

Thirty participants with recently diagnosed Graves’ disease were recruited into the study. Twenty-two participants completed the study of which one participant’s PET/MR image data was of suboptimal quality and was excluded. Hence, twenty-one patients’ data were available for PET/MR-related analysis. Otherwise, twenty-two patients’ data were included. The average duration of treatment required to reach euthyroidism as defined by the achievement of both plasma free thyroxine (FT4) and thyrotropin (TSH) within the normal reference intervals was 28.6 ± 2.3 weeks. Basic measurements of total participants during hyperthyroidism and early euthyroidism states were summarized in Table 1. There was a significant increase in body weight from hyperthyroidism to euthyroidism (P < 0.01), as well as lean mass (P < 0.01) and fat mass (P = 0.02).

**Table 1:** Characteristics of total participants during hyperthyroidism before treatments and early euthyroidism states.

	Hyperthyroidism	Euthyroidism	P-values
BMI (kg/m^2^)	21.8 ± 0.9	23.1 ± 1.0	** < 0.001**
Body weight (kg)	56.0 ± 2.5	59.0 ± 2.8	** < 0.001**
Fat mass (kg)	19.7 ± 1.6	20.6 ± 1.6	**0.02**
Lean mass (kg)	33.8 ± 1.5	35.9 ± 1.8	** < 0.001**
RMR (kcal/day)	1750.0 ± 85.2	1439.0 ± 62.8	** < 0.001**
Fasting glucose (mmol/L)	4.4 ± 0.1	4.5 ± 0.1	0.84
Fasting insulin (µU/mL)	5.6 ± 0.7	6.0 ± 0.8	0.58
HOMA-IR	1.1 ± 0.1	1.2 ± 0.2	0.65
Total cholesterol (mmol/L)	4.5 ± 0.3	5.5 ± 0.3	** < 0.001**
LDL (mmol/L)	2.5 ± 0.2	3.3 ± 0.2	** < 0.001**
HDL (mmol/L)	1.4 ± 0.1	1.7 ± 0.1	** < 0.001**
Triglyceride (mmol/L)	1.4 ± 0.2	1.2 ± 0.2	0.06
VAT (cm^3^)	22.0 ± 2.9	22.4 ± 2.9	0.66
FT3 (pmol/L)	11.5 ± 1.4	5.4 ± 0.3	** < 0.001**
FT4 (pmol/L)	28.5 ± 3.2	12.1 ± 1.3	** < 0.001**
PET SUVmax (g/mL)	1.7 ± 0.2	1.9 ± 0.2	0.52
PET SUVmean (g/mL)	0.9 ± 0.1	0.8 ± 0.1	0.93
MR sFF (%)	72.3 ± 1.4	76.8 ± 1.4	** < 0.001**

N = 22. Data presented as mean ± SEM. P values represents Student’s *t*-test between hyperthyroidism and early euthyroidism, phases. N = 21 for PET SUV, MR FF and VAT data. Statistically significant values are shown in bold. BMI, body mass index, was calculated as body weight (kg) divided by the square of height (m)

*RMR* resting metabolic rate, *HOMA-IR* homeostasis model assessment of insulin resistance, was calculated as fasting glucose*fasting insulin divided by 22.5, *LDL* low-density lipoprotein, *HDL* high-density lipoprotein, *VAT* visceral adipose tissue, *FT3* free triiodothyronine, *FT4* free thyroxine, *PET* positron emission tomography, *MR* magnetic resonance, *SUV* standardized uptake value, *sFF* supraclavicular fat fraction.

The baseline RMR of the participants was significantly higher in the hyperthyroid state compared with the euthyroid state (P < 0.001). There were no significant changes in serum glucose, insulin levels, or HOMA-IR. Serum total cholesterol, LDL-cholesterol, HDL-cholesterol increased significantly from the hyperthyroid to the euthyroid state (P < 0.001). No significant changes were found in triglyceride levels. There were no significant changes in BAT SUVmax and SUVmean from hyperthyroidism to euthyroidism at room temperature. However, there was a significant increase in supraclavicular fat fraction (sFF) from the hyperthyroid to the euthyroid state (72.3 ± 1.4; 76.8 ± 1.4 respectively; P < 0.01).

### Serum amino acids concentration changes during the transition from hyperthyroidism to euthyroidism

Significant reductions were observed in fasting plasma arginine, leucine, methionine, tyrosine as well as valine concentration from hyperthyroid to euthyroid state. Citrulline level was significantly increased. There was no significant changes in total serum amino acid concentration between the two states, whereas BCAA level was significantly reduced (P = 0.01) (Table 2).

**Table 2:** Serum amino acid profile during hyperthyroidism and euthyroidism in participants with Graves’ disease.

	Hyperthyroid status	Euthyroid status	P values
**Amino acids (µmol/L)**			
Alanine	308.4 ± 13.4	330.2 ± 15.0	0.07
Arginine	93.6 ± 2.7	84.1 ± 3.9	**0.04**
Aspartic acid	21.4 ± 2.0	21.8 ± 1.7	0.83
Citrulline	24.5 ± 1.4	27.8 ± 1.8	**0.03**
Glycine	199.4 ± 9.0	201.4 ± 8.9	0.79
Glutamic acid	108.0 ± 6.8	102.1 ± 7.8	0.44
Histidine	75.7 ± 3.0	77.8 ± 2.7	0.46
Isoleucine	57.5 ± 3.1	52.2 ± 2.9	0.15
Leucine	118.5 ± 4.7	104.7 ± 4.9	**0.02**
Methionine	23.4 ± 0.8	20.8 ± 0.8	**0.03**
Ornithine	64.5 ± 4.3	71.2 ± 5.1	0.28
Phenylalanine	65.3 ± 2.1	61.2 ± 2.1	0.11
Proline	145.8 ± 6.6	143.0 ± 6.7	0.64
Serine	102.1 ± 3.5	109.7 ± 3.9	0.11
Tryptophan	47.4 ± 1.9	47.7 ± 2.1	0.89
Tyrosine	62.6 ± 1.8	51.3 ± 2.3	** < 0.01**
Valine	245.2 ± 8.8	214.9 ± 9.4	**0.01**
BCAA	421.2 ± 15.8	371.8 ± 16.8	**0.01**
Total AA	1763.3 ± 50.1	1721.9 ± 55.5	0.47

N = 22. Data presented as mean ± SEM. P values represents Student’s *t*-test between hyperthyroidism and early euthyroidism.

Statistically significant values are shown in bold.

*BCAA* branched-chain amino acids (isoleucine, leucine and valine), *AA* amino acids.

### Amino acids comparison between BAT-positive and BAT-negative participants

We separated the 21 subjects based on BAT status (BAT-positive and BAT-negative subjects) to compare the amino acids difference between BAT-positive and BAT-negative particpants during the hyperthyroid and euthyroid state (Table 3). Participants were divided into two groups: those with detectable ^18^F-FDG uptake with a PET/MR SUV max ≥ 1.5 (BAT-positive; n = 8) and those with no detectable ^18^F-FDG uptake (BAT-negative; n = 13) according to ^18^F-FDG uptake and co-registered with MR FF as per our previous protocol^25^. During the hyperthyroid state, BAT-positive participants had higher BAT SUVmax and SUVmean compared with BAT-negative participants (P < 0.01). sFF was significantly lower in BAT positive compared with BAT-negative participants. These two image parameters supported the separation into the two groups. There was no significant difference in all amino acids levels between BAT-positive and BAT-negative participants in the hyperthyroid state. During the early euthyroid state, BAT-negative participants had significantly higher alanine, aspartic acid, leucine, phenylalanine, BCAA and total AA compared with BAT-positive participants (Table 3). However, if we compare amino acids profile between the hyperthyroid state and euthyroid state in BAT-positive and BAT-negative participants, we found significant reductions in the amino acids above from the hyperthyroid to euthyroid state only in BAT-positive participants but not in BAT-negative participants (Table 4). Taken together, the differences in certain amino acids observed in the euthyroid state between BAT-positive and BAT-negative participants were due to the effects of BAT.

**Table 3:** Comparison amino acids profile between BAT-positive and BAT-negative participants before and after treatment.

	Hyperthyroid state			Euthyroid state		
	BAT-positive	BAT-negative	P-values	BAT-positive	BAT-negative	P-values
SUVmax (g/mL)	2.4 ± 0.3	1.3 ± 0.1	**0.001**	2.1 ± 0.4	1.8 ± 0.3	0.44
SUVmean (g/mL)	1.2 ± 0.1	0.7 ± 0.1	**0.001**	0.9 ± 0.1	0.8 ± 0.1	0.73
sFF (%)	68.1 ± 1.5	74.9 ± 1.8	**0.02**	72.7 ± 1.1	79.3 ± 1.9	**0.02**
Alanine	285.7 ± 23.5	323.9 ± 16.9	0.19	284.6. ± 24.4	361.3 ± 16.0	**0.01**
Aspartic acid	19.0 ± 1.9	23.5 ± 3.1	0.3	17.2 ± 1.7	24.9 ± 2.3	**0.03**
Glutamic acid	100.7 ± 8.0	114.6 ± 10.1	0.35	82.5 ± 10.0	114.2 ± 10.6	0.06
Isoleucine	55.2 ± 3.7	57.4 ± 4.5	0.74	44.5 ± 2.8	56.5 ± 4.2	0.06
Leucine	114.6 ± 6.8	118.6 ± 6.6	0.7	90.0 ± 4.9	114.4 ± 6.7	**0.02**
Valine	246.8 ± 11.7	239.5 ± 12.4	0.7	193.0 ± 11.6	226.3 ± 13.2	0.10
Phenylalanine	63.3 ± 3.6	66.2 ± 2.8	0.54	54.7 ± 2.0	66.1 ± 2.5	**0.01**
Total AA	1714.0 ± 80.0	1804.0 ± 69.0	0.42	1557.0 ± 45.4	1836.7 ± 75.5	**0.01**
BCAA	416.6 ± 20.6	415.5 ± 22.5	0.97	327.6 ± 18.2	397.2 ± 23.6	**0.05**

N = 21. BAT-positive n = 8, BAT-negative n = 13. Data presented as mean ± SEM. P values represents Independent Sample’s *t*-test between BAT-positive and BAT-negative subjects.

Statistically significant values are shown in bold.

*SUVmax* standardized uptake value maximum, *SUVmean* standardized uptake mean value, *sFF* supraclavicular fat fraction, *AA* amino acids, *BCAA* branched-chain amino acids (isoleucine, leucine and valine).

**Table 4:** Comparison amino acids profile between hyperthyroid state and euthyroid state in BAT-positive and BAT-negative participants.

	BAT-positive			BAT-negative		
	Hyperthyroid	Euthyroid	P-values	Hyperthyroid	Euthyroid	P-values
SUVmax (g/mL)	2.4 ± 0.3	2.1 ± 0.4	0.72	1.3 ± 0.1	1.8 ± 0.3	0.17
SUVmean (g/mL)	1.2 ± 0.1	0.9 ± 0.1	0.07	0.7 ± 0.1	0.8 ± 0.1	0.17
sFF (%)	68.1 ± 1.5	72.7 ± 1.1	**0.01**	74.9 ± 1.8	79.3 ± 1.9	** < 0.001**
Alanine	285.7 ± 23.5	284.6. ± 24.4	0.93	323.9 ± 16.9	361.3 ± 16.0	**0.04**
Aspartic acid	19.0 ± 1.9	17.2 ± 1.7	0.46	23.5 ± 3.1	24.9 ± 2.3	0.69
Glutamic acid	100.7 ± 8.0	82.5 ± 10.0	0.07	114.6 ± 10.1	114.2 ± 10.6	0.97
Isoleucine	55.2 ± 3.7	44.5 ± 2.8	**0.04**	57.4 ± 4.5	56.5 ± 4.2	0.87
Leucine	114.6 ± 6.8	90.0 ± 4.9	**0.006**	118.6 ± 6.6	114.4 ± 6.7	0.57
Valine	246.8 ± 11.7	193.0 ± 11.6	**0.004**	239.5 ± 12.4	226.3 ± 13.2	0.34
Phenylalanine	63.3 ± 3.6	54.7 ± 2.0	**0.03**	66.2 ± 2.8	66.1 ± 2.5	0.99
Total AA	1714.0 ± 80.0	1557.0 ± 45.4	**0.05**	1804.0 ± 69.0	1836.7 ± 75.5	0.69
BCAA	416.6 ± 20.6	327.6 ± 18.2	**0.005**	415.5 ± 22.5	397.2 ± 23.6	0.46

N = 21. BAT-positive n = 8, BAT-negative n = 13. Data presented as mean ± SEM. P values represents Independent Sample’s *t*-test between BAT-positive and BAT-negative subjects.

Statistically significant values are shown in bold.

*SUVmax* standardized uptake value maximum, *SUVmean* standardized uptake mean value, *sFF* supraclavicular fat fraction, *AA* amino acids, *BCAA* branched-chain amino acids (isoleucine, leucine and valine).

### Amino acids interactions between BAT and thyroid status

Next, we examined the BAT and thyroid status effects and their interactions on amino acids (Table 5). Serum alanine was significantly associated with BAT status (P = 0.04) but not with patients’ thyroid status or their interaction. Phenylalanine and total AA were also associated with BAT status (P = 0.05). BAT-positive participants had a significantly lower alanine, phenylalanine and total AA levels compared with BAT-negative participants independent of thyroid status. For leucine and BCAA, there were significant associations with patients’ thyroid status (P ≤ 0.01) and a marginally significant interaction (P = 0.06) but not with BAT status. For valine, there was a significant patients’ status effect (P < 0.01) and interaction (P = 0.05) but not for BAT status (P = 0.43). Taken together, leucine, valine and BCAA as a whole were significantly reduced from the hyperthyroid to euthyroid state. The reductions were affected by BAT-status. Interestingly, these reductions were observed only in BAT-positive participants but no differences were found in BAT-negative participants (Table 5).

**Table 5:** Interaction effects between patient status (hyperthyroid and euthyroid states) and BAT status (BAT positive and BAT negative).

	Hyperthyroid		Euthyroid		Patients status effects	BAT status effects	Interaction
	BAT-positive	BAT-negative	BAT-positive	BAT-negative	P values		
SUVmax (g/mL)	2.4 ± 0.3	1.3 ± 0.1	2.1 ± 0.4	1.8 ± 0.3	0.71	**0.008**	0.28
SUVmean (g/mL)	1.2 ± 0.1	0.7 ± 0.1	0.9 ± 0.1	0.8 ± 0.1	0.52	**0.007**	**0.03**
sFF (%)	68.1 ± 1.5	74.9 ± 1.8	72.7 ± 1.1	79.3 ± 1.9	** < 0.0001**	**0.01**	0.90
Alanine	285.7 ± 23.5	323.9 ± 16.9	284.6 ± 24.4	361.3 ± 16.0	0.13	**0.04**	0.11
Aspartic acid	19.0 ± 1.9	23.5 ± 3.1	17.2 ± 1.7	24.9 ± 2.3	0.93	0.06	0.50
Glutamic acid	100.7 ± 8.0	114.6 ± 10.1	82.5 ± 10.0	114.2 ± 10.6	0.25	0.09	0.27
Isoleucine	55.2 ± 3.7	57.4 ± 4.5	44.5 ± 2.8	56.5 ± 4.2	0.13	0.17	0.19
Leucine	114.6 ± 6.8	118.6 ± 6.6	90.0 ± 4.9	114.4 ± 6.7	**0.01**	0.10	0.06
Valine	246.8 ± 11.7	239.5 ± 12.4	193.0 ± 11.6	226.3 ± 13.2	**0.003**	0.43	**0.05**
Phenylalanine	63.3 ± 3.6	66.2 ± 2.8	54.7 ± 2.0	66.1 ± 2.5	0.08	**0.05**	0.08
Total AA	1714.0 ± 80.0	1804.0 ± 69.0	1557.0 ± 45.4	1836.7 ± 75.5	0.30	**0.05**	0.12
BCAA	416.6 ± 20.6	415.5 ± 22.5	327.6 ± 18.2	397.2 ± 23.6	**0.007**	0.24	0.06

N = 21. BAT-positive n = 8, BAT-negative n = 13. Data presented as mean ± SEM. The patients’ status effects, BAT status effects and interactions between patients’ status and BAT status were tested using linear mixed effects model adjusted by BMI.

Statistically significant values are shown in bold.

*SUVmax* standardized uptake value maximum, *SUVmean* standardized uptake mean value, *sFF* supraclavicular fat fraction, *AA* amino acids, *BCAA* branched-chain amino acids (isoleucine, leucine and valine).

### Relationship of BCAA and metabolic parameters

**Figure 1:**
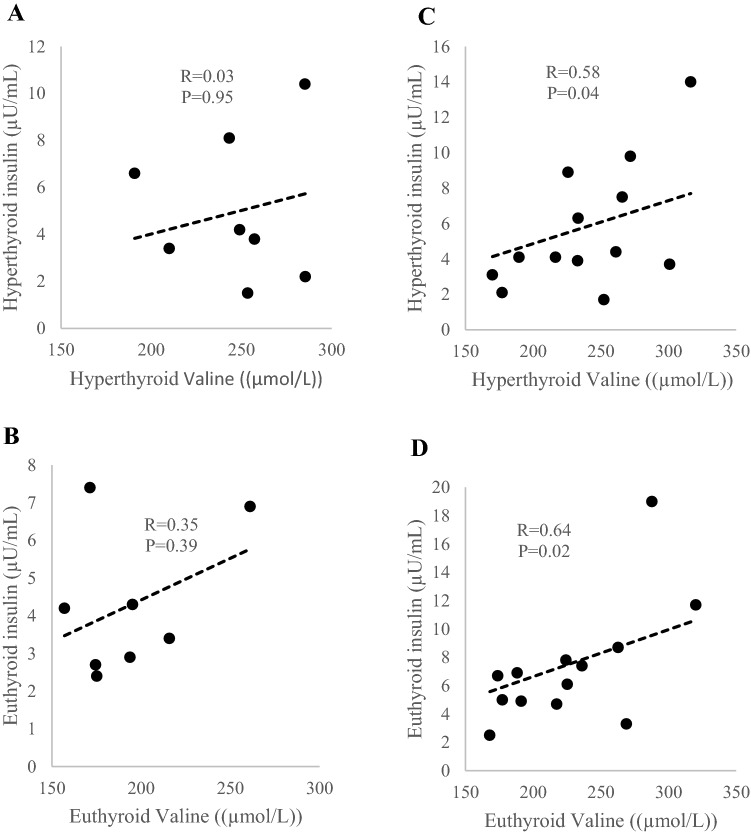
Correlations between valine concentration with insulin concentration at hyperthyroid state (**A)** and euthyroid state (**B)** in BAT-positive subjects and BAT-negative subjects (**C,D)**. n = 8 in BAT-positive subjects; n = 13 in BAT-negative subjects.

**Figure 2:**
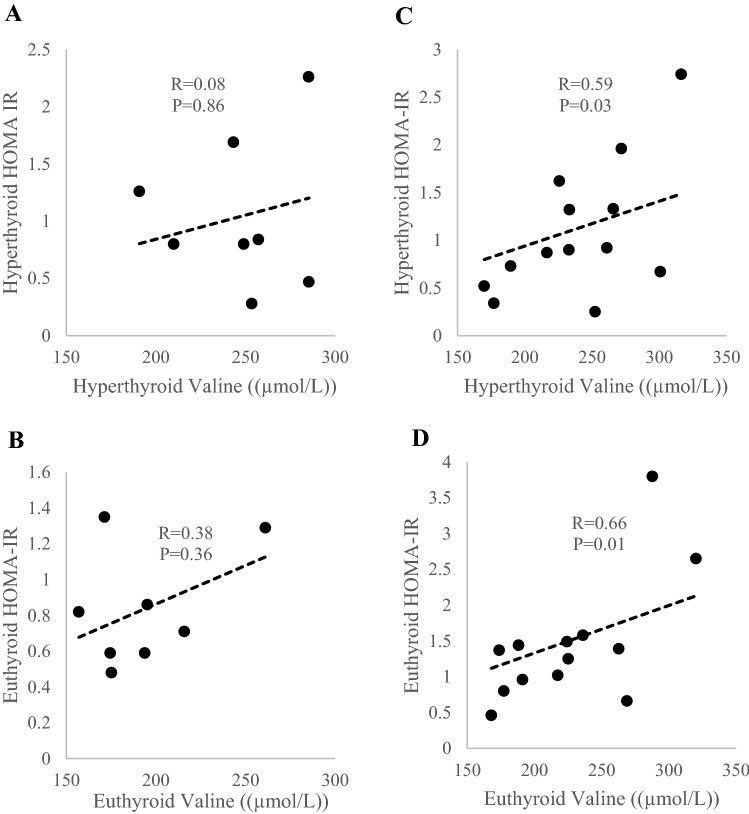
Correlations between valine concentration with HOMA-IR score at hyperthyroid state (**A)** and euthyroid state (**B)** in BAT-positive subjects and BAT-negative subjects (**C,D)**. n = 8 in BAT-positive subjects; n = 13 in BAT-negative subjects.

There was a positive association between serum valine level and sFF in the hyperthyroid state (r = 0.46, P = 0.04), but not in the euthyroid state (Table 6). During the hyperthyroid state, valine but not leucine and isoleucine was positively associated with FT3 (r = 0.50, P = 0.02), RMR (r = 0.45, P = 0.03), insulin (r = 0.45, P = 0.04), HOMA-IR (r = 0.48, P = 0.03) and VAT (r = 0.45, P = 0.04). During the euthyroid state, valine, as well as leucine and isoleucine were positively associated with many metabolic parameters we have listed in Table 6. However, no significant relationship was found between valine and FT3 in the euthyroid state. Since the AA profile changes were different between BAT-positive and BAT-negative patients, we next correlated insulin and HOMA-IR with valine separately in BAT-postive and BAT-negative patients. Valine was significantly positively correlated with insulin level in BAT-negative participants in both hyperthyroid (r = 0.58, P = 0.04) and euthyroid states (r = 0.64, P = 0.02) (Fig. 1). There were no correlation between insulin and valine in BAT-positive participants. Valine was significantly positively correlated with HOMA-IR score in BAT-negative participants in the hyperthyroid state (r = 0.58, P = 0.04) and euthyroid state (r = 0.64, P = 0.02). No significant correlations were found between valine and HOMA-IR in BAT-positive participants (Fig. 2).

**Table 6:** Correlations between BCAA concentration at hyperthyroid and euthyroid state with metabolic parameters at both state.

	Hyperthyroid state			Euthyroid state		
	Valine	Leucine	Isoleucine	Valine	Leucine	Isoleucine
SUVmax (g/mL)	0.29 (0.20)	0.05 (0.85)	0.12 (0.62)	0.04 (0.87)	0.01 (0.96)	−0.13 (0.57)
SUVmean (g/mL)	0.27 (0.25)	0.18 (0.43)	0.11 (0.65)	0.25 (0.28)	0.25 (0.28)	0.04 (0.85)
sFF (%)	**0.46 (0.04)**	0.33 (0.14)	0.21 (0.16)	0.35 (0.12)	**0.43 (0.05)**	0.33 (0.15)
FT3 (mmol/L)	**0.50 (0.02)**	0.40 (0.07)	0.30 (0.17)	−0.10 (0.65)	−0.08 (0.72)	−0.20 (0.38)
RMR (kcal/day)	**0.45 (0.03)**	0.31 (0.17)	0.36 (0.10)	**0.41 (0.06)**	0.33 (0.13)	0.26 (0.23)
Insulin ( (µU/mL))	**0.45 (0.04)**	0.32 (0.15)	**0.42 (0.05)**	**0.60 (0.003)**	**0.69 (0.001)**	**0.68 (0.001)**
HOMA IR	**0.48 (0.03)**	0.29 (0.19)	0.40 (0.07)	**0.62 (0.002)**	**0.69 (0.001)**	**0.69 (0.001)**
VAT (cm^3^)	**0.45 (0.04)**	0.29 (0.19)	0.25 (0.26)	**0.63 (0.002)**	**0.60 (0.004)**	**0.53 (0.01)**
Fat mass (kg)	0.26 (0.25)	−0.01 (0.96)	0.01 (0.98)	**0.51 (0.02)**	0.41 (0.06)	**0.47 (0.03)**
Lean mass (kg)	0.33 (0.14)	0.27 (0.22)	0.41 (0.06)	**0.60 (0.003)**	**0.60 (0.003)**	**0.46 (0.03)**
Cholesterol (mmlo/L)	0.23 (0.31)	0.20 (0.38)	0.25 (0.27)	**0.51 (0.02)**	**0.59 (0.004)**	**0.45 (0.04)**
Triglyceride (mmol/L)	0.30 (0.17)	0.18 (0.42)	0.29 (0.19)	**0.71 (< 0.001)**	**0.82 (< 0.001)**	**0.77 (< 0.001)**
HDL (mmol/L)	−0.27 (0.22)	−0.19 (0.40)	−0.18 (0.43)	−0.31 (0.16)	v0.30 (0.18)	−0.29 (0.20)

N = 22 except for SUV max, SUV mean, sFF and VAT data (N = 21). Correlations were used to assess relationship between variables. Valine, leucine and isoleucine at hyperthyroid state correlations with metabolic parameters at hyperthyroid state. Valine, leucine and isoleucine at euthyroid state correlations with metabolic parameters at euthyroid state. Significant correlations between variables are shown in bold with corresponding coefficient (r) and P values.

*SUVmax* standardized uptake value maximum, *SUVmean* standardized uptake mean value, *sFF* supraclavicular fat fraction, *FT3* free triiodothyronine, *RMR* resting metabolic rate, *HOMA-IR* homeostasis model assessment of insulin resistance, was calculated as fasting glucose*fasting insulin divided by 22.5, *VAT* visceral adipose tissue, *HDL* high-density lipoprotein.

## Discussion

Hyperthyroidism is a hypermetabolic state leading to metabolic changes with increased energy expenditure (EE) and thermogenesis. Thyroid hormone plays an important role in EE, body composition, insulin sensitivity, amino acid (AA) level and BAT development and function in hyperthyroid state. BAT is involved in the regulation of EE and metabolic syndrome. AA, especially BCAA, is often beneficial to EE but paradoxically associated with obesity and diabetes when elevated. It seems there are complex interrelationships among thyroid hormone, BCAA and BAT function. In our current study, we examined AA, especially BCAA changes, during the transition from the hyperthyroid to euthyroid state and the relationship between BCAA and BAT. Our key findings were: (1) certain AA, especially BCAA, were decreased from hyperthyroid to euthyroid state; (2) the reductions of AA and BCAA were found only in BAT-positive participants; (3) serum valine concentration was positively associated with insulin sensitivity in BAT-negative participants independent of thyroid status.

From our precious published data^26^, we have found that lean mass was decreased in the hyperthyroid state and it was significantly increased when they achieved euthyroidism (from 33.8 ± 1.5 to 35.9 ± 1.8 kg) which was consistent with another published paper^27^. The thyroid hormone effects on lean mass are probably due to protein turnover (protein catabolism and synthesis)^7^. Without labeled isotope amino acid tracers, it would not be possible to conclude definitively the dynamics of BCAA release from muscle breakdown compared with the rate kinetics of BCAA uptake by active BAT. It is possible that the uptake of BCAA by activated BAT might reduce the known anabolic effect of BCAA on skeletal muscles. Hence, the loss of lean mass is expectedly greater during hyperthyroidism among BAT-positive individuals compared with those who are BAT-negative. From our dataset, there was no significant difference in serum creatinine between BAT-positive and BAT-negative groups within each hyperthyroid and euthyroid state. There was however a significant increase of serum creatinine level when transitioning from hyperthyroidism and euthyroidism state, an observation that implies the role of thyroid hormones rather than BAT mediating this effect. Taken together, based on our current study, we did not find any association between BAT and protein breakdown during the transition from hyperthyroidism to euthyroidism. As well, certain AAs release may vary during the transition from the hyperthyroid to euthyroid state. It was reported that elevated tyrosine was associated with increased protein breakdown^7^. We have found that serum tyrosine concentration was higher in hyperthyroidism compared with euthyroidism which probably implies more protein breakdown and lower lean mass during hyperthyroidism. The significant decrease of tyrosine was consistent with a previous study in the Asian population^28^ which suggested protein breakdown was associated with certain AAs release.

In our study, BCAA (leucine, isoleucine and valine) were significantly higher in hyperthyroidism compared with euthyroidism, especially valine and leucine in all 22 participants including both males and females. However, Chng et al.^28^ reported BCAA did not change after achieving euthyroidism in Chinese women in Singapore. We initially thought that perhaps the slightly different gender and ethnic proportions may have contributed to this inconsistency. However, even when we examined only Chinese female participants (n = 16), BCAA was significantly decreased after treatment (data not shown). In order to understand the difference between these two similar studies, we separated the participants to BAT-positive and BAT-negative groups based on BAT activation as reported in our previous studies^23,25^. Surprisingly, we found that BCAA did not change after treatment in BAT-negative participants. However, BCAA, phenylalaninie and total AA were significantly decreased after treatment in BAT-positive participants. Taken together, we hypothesise that BAT activation is the main reason that accounted for the inconsistent reports of BCAA changes during the transition from hyperthyroidism to euthyroidism by different researchers. Due to more BAT-negative patients in the cohort study, less net changes in BCAA were found if we did not separate the participants by BAT status. Hence, our results suggest that BAT may oxidise BCAA during the transition from hyperthyroidism to euthyroidism.

Epidemiological studies reported that elevated BCAA levels are positively associated with obesity, insulin resistance and diabetes in humans^29–31^. Yoneshiro et al.^10^ recently reported cold-induced BAT activation utilized BCAA in the mitochondria for thermogenesis and promoted BCAA clearance in mice and humans which is mediated by SLC25A44. SLC25A44 is an amino acid transporter most expressed in the mitochondria of BAT. This study also found serum valine level was significantly decreased in cold-induced BAT healthy subjects. In our study, we found that the decrease in serum valine concentration after treatment was affected by BAT status. During early hyperthyroid state, valine concentration (rather than leucine and isoleucine in BCAA) was significantly associated with FT3, EE, insulin, HOMA-IR and VAT. These findings demonstrate that valine in BCAA is the main amino acid associated with insulin resistance and thyroid dysfunction in hyperthyroidism.

It was reported that insulin resistance and diabetes was not only associated with elevated BCAA but also with increased circulating level of 3-hydroxyisobutyrate (3-HIB), a catabolic intermediate of valine^32,33^. Nilsen et al. also^34^ reported circulating 3-HIB plays a novel role in modulating white and brown adipocyte metabolism. These data suggested elevated circulating valine and its degradation product 3-HIB play an important role in insulin resistance in humans. Whether 3-HIB was elevated in hyperthyroid patients needs to be explored in future. Meanwhile, leucine, isoleucine and valine were strongly positively associated with insulin, HOMA-IR and VAT after treatment in early euthyroidism. BCAA was strongly positively associated with fat mass, lean mass, cholesterol and triglyceride levels in euthyroidism but not in hyperthyroidism. Serum valine and other BCAAs play a very different role in different thyroid status. Short-term reduction of dietary BCAAs reduced postprandial insulin secretion and improved white adipocyte metabolism^12^. Therefore, increased BCAA supplementation should be cautiously recommended even in healthy normal people. The underlying mechanism mediating the different effects via thyroid hormones remains unknown.

Interestingly, after we separated the participants to BAT-positive and BAT-negative groups, we found that the strongly positive associations only exist in BAT-negative participants. Associations of valine with insulin resistance (insulin and HOMA-IR) were lost in BAT-positive participants in both hyperthyroid and euthyroid state. These observations caught our attention because BAT seemingly induced a vital difference on the effects of valine on insulin resistance. These data suggested elevated circulating valine and its degradation product 3-HIB probably play an important role in insulin resistance in humans independent of BAT status. That said, unlike plasma levels of valine which correlated with insulin and HOMA-IR in BAT-negative patients, the decline in valine levels occurs only among BAT-positive patients. As BAT mitigates against the effects of valine on insulin and HOMA-IR, therefore valine did not correlate with insulin level and HOMA-IR in BAT-positive patients.

In our current study, there are some limitations. First, in our design, we hypothesise that thyroid hormones elevated in hyperthyroidism may induce BAT activity. Therefore, we evaluated BAT activity at normal room temperature conditions without resorting to cold exposure typically utilized to stimulate BAT. However, SUVmean values were rather low without cold exposure. It turned out that BAT activity under this circumstance was lower compared with cold-induced BAT activation in our previous studies in healthy subjects^25,35,36^. Hence, even small amounts of active BAT protect against elevated BCAA-induced insulin resistance and alter metabolism significantly. FDG/PET with cold exposure may have high accuracy and reliability to assess inter- and intra-individual fluctuations of BAT activity. Secondly, our sample size was small as was the number of BAT-positive participants in our study. During the data analysis, it is noteworthy that our multiple comparisons are largely exploratory in nature rather than multiple hypotheses testing per se. Hence, we have used the actual p-values without Bonferroni correction for multiple testing to prevent over correction which could result in the premature dismissing of potentially important relationships in exploratory research. Many of the reported variables are associated with each other and the final findings are in the same direction which strengthens our confidence in the results. The conclusion from our results thus needs further validation in larger cohorts to elucidate the mechanisms of how active BAT protects against elevated BCAA-induced insulin resistance.

In conclusion, our results demonstrated that active BAT likely plays an important role in reducing circulating blood BCAA levels in hyperthyroid patients. Based on Yoneshiro et al.^10^ and our work, we proposed that active BAT protects against the development of insulin resistance via stimulating BCAA catabolism in hyperthyroid patients. Therefore, though still largely preliminary, our data suggest that active BAT not only serves as a metabolic substrate for glucose and fatty acids but also for BCAAs. Further larger scale longitudinal studies can be conducted to confirm our results. Applying cold stimulation and other nutriceutical supplementation such as capsinoids to stimulate BAT or browning of white adipose tissue may help combat metabolic disease and improve metabolic health.
